# Advancements in Assessing Pathological Fracture Risk: News on Mirels’ Score

**DOI:** 10.3390/cancers17060973

**Published:** 2025-03-13

**Authors:** Cesare Meschini, Alessandro El Motassime, Omar El Ezzo, Antonio Ziranu, Giulio Maccauro, Raffaele Vitiello

**Affiliations:** 1 Department of Orthopedics and Geriatric Sciences, Catholic University of the Sacred Heart, 00168 Rome, Italy; cesare.meschini01@icatt.it (C.M.); giulio.maccauro@policlinicogemelli.it (G.M.); raffaele.vitiello@policlinicogemelli.it (R.V.); 2 Department of Orthopedics and Rheumatological Sciences, Fondazione Policlinico Universitario A. Gemelli IRCCS, 00168 Rome, Italy; omar.elezzo@guest.policlinicogemelli.it; 3 Orthopedics and Traumatology, Università Cattolica Del Sacro Cuore, 00168 Roma, Italy; antonio.ziranu@policlinicogemelli.it; 4 Ospedale Isola Tiberina—Gemelli Isola, Gemelli Isola, 00186 Roma, Italy

**Keywords:** Mirels’, oncologic, femur fracture, pathologic, score, impending

## Abstract

The Mirels’ score is a widely utilized tool for assessing the risk of pathological fractures in patients with metastatic bone disease. This scoring system assists clinicians in estimating fracture likelihood based on clinical, radiological, and anatomical parameters, thereby guiding treatment decisions and management strategies. This review provides a comprehensive analysis of the Mirels’ score, detailing its development, clinical applications, and its role in predicting fractures across various metastatic cancers. Additionally, it discusses the score’s limitations, including its potential to over- or underestimate fracture risk, and explores recent advancements in imaging and biomarker technologies that may enhance or complement its predictive accuracy. By critically examining the existing literature, this review aims to offer a thorough understanding of the Mirels’ score’s strengths and weaknesses, while considering its future role in personalized cancer care.

## Simple Summary

Pathological fractures in long bones, especially those involving primary or metastatic tumors, significantly impact patients, both physically and psychologically. Tumors often target the long bones, which are also common sites for metastasis. The recovery from these fractures deviates from the normal healing processes due to the tumor’s interference with bone repair, often requiring surgical intervention. Various methods have been developed to assess fracture risk, with Mirels’ scoring system being widely used. This system evaluates factors such as bone site, lesion characteristics, matrix density, and pain. While effective, the system’s low specificity leads to false positives. Studies have explored modifications and alternatives to improve accuracy, such as CT-based structural rigidity analysis (CTRA) and finite element analysis (FEA), which outperform the Mirels’ score in predicting fractures. Additionally, new scoring systems focusing on factors like lesion size and location offer enhanced sensitivity. However, despite these advances, the Mirels’ score remains central in clinical decision making for managing impending fractures.

## 1. Introduction

Pathological fractures in the major long bones can have a significant impact on patients, both physically and psychologically, especially when they involve primary or secondary tumors. Musculoskeletal tumors often target the long bones, which are also common sites for metastatic spread. In fact, bones are the third most common site for metastasis, following the lungs and liver [1,2]. Furthermore, this fracture type deviates from the standard healing processes [3] due to the presence of a tumor at the fracture site, which interferes with bone healing. As a result, the majority of these fractures are effectively managed through surgical intervention [4].

Pathological fractures typically manifest with early pain and an injury mechanism that does not align with the nature of the injury. Impending fractures are frequently detected on radiographs when assessing hip pain or during staging or monitoring after prior injuries [5]. This symptom is, in fact, very important for the assessment and management of this patient population, as it may serve as an early warning signal warranting further investigation. However, pain must be properly evaluated in relation to the analgesic therapy that these patients typically receive.

Over the years, various authors have developed methods to assess the risk of actual fractures, and some of these methods are still in use today. One of the first authors to establish guidelines for managing impending fractures was Harrington in 1986, who introduced specific criteria and recommended prophylactic stabilization for metastatic or equivalent lesions with lytic characteristics, a size greater than half the bone’s diameter or 2.5 cm, or associated with persistent pain or radiographic progression after radiation therapy. However, the reliability and reproducibility of these guidelines have been questioned [6]. Additionally, significant importance continues to be placed on the scoring system described by Mirels in 1989 [7].

Several years later, Mirels [7] conducted a study with the aim of evaluating the various risk factors outlined in the literature, as well as testing the hypothesis that a weighted scoring system could provide greater accuracy than any single risk factor in predicting impending pathologic fractures. Drawing from the available literature at the time, Mirels incorporated four key parameters into his scoring system: the affected long bone, the situs of the metastatic lesion, the density of the matrix, and the presence of pain. Each criterion is assigned a score ranging from 1 to 3, with higher scores indicating an elevated likelihood of fracture (Table 1). A score exceeding 7 denotes a prelude to fracture (≥8), according to Mirels’ classification. The study found a Mirels’ score under 7 indicates a low fracture risk of 4%, with a 22% false-positive rate, suggesting that patients avoid prophylactic fixation. A score of 8 was linked to a 15% fracture risk and a 6% false-positive rate. Additionally, a score of 9 showed a higher fracture risk of 33%, with no false positives. Thus, lesions scoring 9 or above require prophylactic surgical stabilization [8]. Notably, this system possesses limitations; while its sensitivity is relatively high (71–100%), its specificity remains low (13–50%). Such a discrepancy may precipitate unnecessary treatments for these patients, thereby leading to associated complications [9].

Mirels’ score has long been a critical factor in deciding whether to prophylactically treat an impending fracture. Over time, various authors have published papers aimed at improving Mirel’s score. The purpose of this paper is to provide an overview of their proposals and to explore these advancements.

## 2. Materials and Methods

A review was performed covering literature up until January 2025. A comprehensive search for research articles investigating the Mirels’ score and its applications was conducted across three prominent online databases: Medline, Web of Science, and Scopus. Only articles published in English were considered for inclusion. Additionally, the reference lists of the selected studies were examined to identify other potentially relevant articles that may have been overlooked in the initial database search. The final set of studies included longitudinal research (both retrospective and prospective) and randomized controlled trials. Exclusion criteria were applied to filter out case reports, expert opinions, previous systematic reviews, letters to the editor, and studies that did not directly pertain to the review’s objectives.

The screening process began with two independent reviewers evaluating titles and abstracts. Full-text articles were retrieved for any studies meeting the inclusion criteria or those that presented ambiguities. Following this, each study was independently assessed by two reviewers for compliance with the inclusion criteria, with any discrepancies resolved through consultation with the senior author. Relevant data, including information on the Mirels’ score, its application, and any proposed modifications or alternative assessment methods, were extracted from each study.

## 3. Discussion

### 3.1. Mirels’ Validity and Reproducibility

Numerous authors have examined the validity and reproducibility of the score, also assessing inter- and intra-observer variability. In 2003, Damron et al. [10] undertook a critical assessment of Mirels’ rating system for predicting impending pathologic fractures of the femur, specifically evaluating its reproducibility, validity, and applicability across examiners with varying levels of experience and training. Their analysis suggested that, while the Mirels’ score appeared to be the most reliable system at that time, outperforming clinical judgment across different experience levels, there was a need for more specific parameters to improve its accuracy and clinical utility.

Mac Niocaill et al. [11] also conducted a study to evaluate the inter- and intra-observer variability of this score. This study was conducted with the exclusion of pain to minimize subjective bias, thereby focusing on the radiological components of the scoring system. The findings revealed moderate to good inter-observer and intra-observer agreement. Agreement was the strongest for the lesion site and exhibited greater variability concerning the size and nature of the lesions. These findings imply that the Mirels’ system is both reliable and repeatable to assess fracture risk in metastatic bone lesions, thereby supporting its continued application in clinical practice.

The validity and reproducibility of the score have also been assessed for the lower limb. In 2018, Howard et al. [12] evaluated a sample of 62 patients. Radiographic images were assessed by four investigators utilizing the Mirels’ system, emphasizing the objective components (site, size, and radiographic characteristics), while excluding pain, according to the methods used by Mac Niocaill in 2011. According to their findings, the Mirels’ score demonstrates insufficient reproducibility and should be employed with caution in clinical practice, suggesting that more dependable and objective instruments, such as CT-based structural rigidity analysis (CTRA), may provide enhanced prediction of fracture risk.

### 3.2. What Is New for the Upper Limb

The Mirels’ scoring system was further questioned regarding its validity and reproducibility for humeral lesions. Specifically, Evans et al. [13] hypothesized that due to the differences in load-bearing demands between the upper and lower limbs, the Mirels’ score may not be as accurate for predicting impending fractures of the humerus. They aimed to assess whether the Mirels’ score could more accurately identify impending humeral fractures than could clinical judgment alone. They involved 72 physicians from various specialties and/or experience levels to assess 16 anonymous case histories. Their results highlighted that the Mirels’ score was reproducible and despite its low specificity, it remained the preferred system for evaluating metastases in long bones. Additionally, the study identified that a score of 7 or higher was optimal for predicting impending pathological fractures of the humerus, maintaining comparable specificity and sensitivity to those observed for other long bones.

This data was retrieved from Hoban et al. [14]. In 2022, they evaluated the Mirels’ scoring system for metastatic bone lesions of the upper limb, determining that the sensitivity and specificity for upper limb lesions were inadequate when using the standard cutoff of 9/12, with sensitivity recorded at 14% and specificity at 73%. Adjusting the threshold to 7/12 resulted in an improved sensitivity of 63%, albeit at the expense of specificity, which decreased to 55%. Additionally, the study reported moderate to substantial intra-observer and inter-observer reliability for the Mirels’ score. Hoban et al. concluded that the Mirels’ score is not accurate enough for predicting pathologic fractures in upper limb metastatic lesions and as previously proposed by Evans, recommended employing a cutoff of 7/12 for considering prophylactic fixation, enhancing sensitivity for fracture prediction. They further suggest that a more sophisticated scoring system, specifically tailored to upper limb lesions, may be necessary to enhance clinical decision making.

### 3.3. Mirels’ Score and New Technologies

As an established scoring system primarily based on X-ray evaluation, several comparisons have been made between the Mirels’ score and the use of more advanced technologies.

In the study conducted by Damron et al. in 2016 [15], a comparison was made between CT-based structural rigidity analysis (CTRA) and the Mirels’ scoring system. The first technique demonstrated superior sensitivity (100% compared to 66.7%), specificity (61% versus 47.9%), and predictive values when contrasted with Mirels’ scoring. Furthermore, ROC curve analysis revealed that CTRA was associated with greater accuracy, yielding a “good” accuracy (AUC = 0.801) in comparison to the “poor” accuracy of Mirels’ at a score of ≥9 (AUC = 0.56). Following the adjustment for variables, such as age and lesion size, CTRA was recognized as a more dependable predictor of fracture risk. According to the findings, CTRA is a more effective method for evaluating fracture risk in femoral metastatic disease, thereby potentially enhancing clinical decision making compared to the use of traditional Mirels’ scoring.

A pilot study conducted by Riaz et al. in 2018 [16] proposes a modified version utilizing 99mTc MDP SPECT–CT. The study aimed to evaluate the effectiveness of SPECT–CT in comparison to conventional X-ray-based Mirels’ scoring. The results indicated that SPECT–CT was more sensitive in detecting cortical lysis, identifying 24% of cases compared to just 6.3% found with X-rays. Additionally, SPECT–CT provided new parameters, such as circumferential involvement and extra-osseous soft tissue involvement, which were not assessed by X-rays. The modified Mirels’ score was statistically significantly different from the conventional score, especially for lesions with scores near the cutoff for surgery. The study suggests that SPECT–CT may improve fracture risk prediction in skeletal metastases, offering more accurate assessment for treatment decisions, especially in cases where the traditional Mirels’ score is ambiguous.

Kaupp et al. [17] conducted an analysis that involved a comparative evaluation of Mirels’ scoring against two advanced techniques: CT-based structural rigidity analysis (CTRA), as proposed by Damron in 2016, and finite element analysis (FEA). The study elucidates the limitations inherent in Mirels’ scoring, particularly its low specificity, and illustrates that both CTRA and FEA yield more precise predictions regarding fracture risks. Both CTRA and FEA exhibited superior accuracy in regards to fracture prediction relative to Mirels’ scoring, albeit neither technique is without its limitations. Despite their numerous advantages, these sophisticated methodologies, especially FEA, have not yet attained extensive implementation in clinical environments due to their complexity and requisite resources. The authors concluded by asserting that while Mirels’ scoring continues to be employed frequently, CTRA and FEA represent promising advancements in fracture prediction for metastatic bone disease, with CTRA emerging as a more viable option.

### 3.4. What Changes Have Been Proposed?

Several attempts have been made over the years to implement and enhance the Mirels’ score, exploring various approaches.

In 2021, Toci et al. [18] aimed to develop a novel scoring system designed to predict pathological fractures in patients suffering from multiple myeloma-related long-bone lesions. They included in the new scoring system, as significant predictors of fracture, lesion size and latency; location, i.e., whether it was in the metaphysis or other regions; and presence of pain. The newly established scoring system exhibited a higher sensitivity of 69% in comparison to the Mirels’ score. Despite the enhanced sensitivity of the new scoring system, its performance regarding specificity, PPV, and NPV remained comparable to that of Mirels’ scoring. The study concludes that the newly proposed scoring system may present a more sensitive instrument for identifying patients at risk of pathologic fractures due to multiple myeloma and could help in determining which patients require further orthopedic evaluation or advanced imaging.

Crenn et al. [19] conducted an analysis of the factors influencing the decision to operate on patients with long-bone metastasis at the fracture stage as opposed to implementing preventive measures, utilizing the Mirels’ scoring system. Four independent predictive factors for surgery at the fracture stage were identified: advanced age, elevated pain scores (VAS > 6), WHO grade greater than 2, and the upper-limb location of metastasis. The authors decided to modify the pain assessment, the element with the highest degree of inter-observer discordance, by using the VAS, which is less subjective compared to Mirels’ criteria. The authors proposed that these factors, in conjunction with the original Mirels’ criteria, may enhance the accuracy of fracture risk predictions.

The results underscore the limitations of the Mirels’ score, particularly its inadequacy in accurately distinguishing between various fracture-risk stages. They concluded by suggesting the integration of additional clinical factors, with no additional costs, to improve its predictive validity.

Amendola et al. (2023) [20] present a computational methodology to enhance the Mirels’ score by revising the location component, with a precise focus on the proximal femur, incorporating an in-depth biomechanical analysis through finite element modeling. By simulating lytic lesions at 32 designated sites in the proximal femur, the authors developed a location-based strength fraction that more accurately predicts fracture risk, based on the placement of lesions. This study illustrates that the amended Mirels’ score significantly improved the accuracy of fracture prediction, achieving a higher area under the receiver operating characteristic (ROC) curve (AUC = 0.95) in comparison to the original Mirels’ score (AUC = 0.85). Furthermore, decision curve analysis showed that the modified system diminished false positives by 17–20%, thereby enhancing clinical decision-making capabilities.

Desai et al., in 2024 [21], conducted an internal validation of the modified Mirels’ scoring system introduced by Amendola the previous year, corroborating its enhanced predictive accuracy. This validation study achieved its goal of corroborating the conclusions of Amendola’s paper, demonstrating that the modification in scoring proposed by their group the year before enhances sensitivity and specificity, leading to a reduction in unnecessary surgery, while preserving its accuracy in predicting fractures. The efficacy of this modified Mirels’ scoring system in improving the prediction of pathologic fractures in metastatic femoral lesions is highlighted by both studies. Integrating computational analysis alongside validation studies confirms that the proposed modification improves specificity, with no reduction in sensitivity, making it a more effective instrument for clinical practice. Table 2.

## 4. Conclusions

This article provides a comprehensive review of the recommendations made by various authors regarding the implementation and improvement of the Mirels’ scoring system. Initially, this score was identified as a good method for evaluating the treatment of bone lesions, and numerous authors confirmed its validity and reproducibility. Despite its continued utility as a simple method that does not require advanced technology, the Mirels’ score exhibits inherent limitations, such as moderate sensitivity and low specificity. Consequently, several authors have proposed various strategies to modify or enhance its accuracy. For instance, two authors have suggested adjusting the cutoff for treatment in the upper limb, thereby increasing the score’s efficacy for such lesions. The use of advanced technologies, such as CTRA and 99mTc MDP SPECT–CT, offers a more accurate approach for fracture management; however, their accessibility remains a challenge, as they are not readily available to all patients. The incorporation of easily applicable new parameters has enhanced the sensitivity and specificity of the Mirels’ score, with a particular focus on pain, which is a critical symptom in assessing impending fractures. The recent literature, along with the authors of this paper, agree on the need to refine this scoring system in an evolving healthcare environment, driven by the emergence of new technologies and more precise methodologies.

## Figures and Tables

**Table 1:** Mirels’ scoring system.

Parameter Score	1	2	3
Site	Upper limb	Lower limb	Peritrochanteric
Pain	Mild	Moderate	Severe
Lesion	Blastic	Mixed	Lytic
Size	<1/3	1/3–2/3	>2/3

**Table 2:** Synthesis of the main concepts of the discussed articles.

First Author	Year	N° Patients	Main Concepts
Damron [10]	2003	12	Mirels’ score appeared to be the most reliable system at that time, but needs more specific parameters to improve its accuracy and clinical utility.
Evans [13]	2008	16	Proposed a score of 7/12 as cutoff for treatment in humeral lesions, with comparable specificity and sensitivity to those observed for other long bones.
Mac Niocaill [11]	2011	28	Mirels’ system is both reliable and repeatable to assess fracture risk in metastatic bone lesions.
Damron [15]	2016	125	CTRA is a more effective method for evaluating fracture risk in femoral metastatic disease.
Riaz [16]	2018	16	Proposed to asses metastatic lesions with 99mTc MDP SPECT–CT: this technique offers a more accurate assessment for treatment decisions.
Howard [12]	2018	62	Mirels’ score demonstrates insufficient reproducibility and should be employed with caution in clinical practice.
Crenn [19]	2020	245	Suggested the integration of additional clinical factors: advanced age, elevated pain scores (VAS > 6), WHO grade greater than 2, and the upper-limb location of metastasis.
Toci [18]	2021	163	Proposed a new scoring system for multiple myeloma-related long-bone lesions that presents a more sensitive instrument.
Kaupp [17]	2021	7	CTRA and FEA represent promising advancements in fracture prediction for metastatic bone disease.
Hoban [14]	2022	45	Recommended employing a cutoff of 7/12 for upper limb lesions.
Amendola [20]	2023	48	Modified Mirels’ scoring considering only the proximal femur, utilizing a computational methodology. The modified system diminished false positives by 17–20%.
Desai [21]	2024	40	Validation of the modification proposed by Amendola. Greater sensitivity and specificity of the modified score using computational analysis.

## Data Availability

No new data were created in this study.

## Abbreviations

The following abbreviations are used in this manuscript:

CTRA	CT-based structural rigidity analysis
FEA	Finite element analysis

## References

[1] Pesare E., Meschini C., Caredda M., Messina F., Rovere G., Solarino G., Ziranu A. (2024). Carbon vs. Titanium Nails in the Treatment of Impending and Pathological Fractures: A Literature Review. J. Clin. Med..

[2] El Motassime A., Meschini C., Di Costa D., Rovere G., Matrangolo M.R., De Maio F., Farsetti P., Ziranu A., Maccauro G., Vitiello R. (2023). Functional Outcomes and Shoulder Instability in Reconstruction of Proximal Humerus Metastases. Curr. Oncol..

[3] Ghimire S., Miramini S., Edwards G., Rotne R., Xu J., Ebeling P., Zhang L. (2020). The investigation of bone fracture healing under intramembranous and endochondral ossification. Bone Rep..

[4] Kong A.C., Zarate S.D., Belzarena A.C. (2021). Missed pathological femoral neck fracture undergoes spontaneous healing. Radiol. Case Rep..

[5] Verspoor F.G.M., Hannink G., Parry M., Jeys L., Stevenson J.D. (2023). The Importance of Awaiting Biopsy Results in Solitary Pathological Proximal Femoral Fractures: Do We Need to Biopsy Solitary Pathological Fractures?. Ann. Surg. Oncol..

[6] Harrington K.D. (1988). Orthopaedic Management of Metastatic Bone Disease.

[7] Mirels H. (1989). Metastatic disease in long bones. A proposed scoring system for diagnosing impending pathologic fractures. Clin. Orthop. Relat. Res..

[8] Hershkovich O., Sakhnini M., Barkay G., Liberman B., Friedlander A., Lotan R. (2023). Femoral metastatic pathological fractures, impending and actual fractures—A patient survival study. Surg. Oncol..

[9] Damron T.A., Mann K.A. (2020). Fracture risk assessment and clinical decision making for patients with metastatic bone disease. J. Orthop. Res..

[10] Damron T.A., Morgan H., Prakash D., Grant W., Aronowitz J., Heiner J. (2003). Critical evaluation of Mirels’ rating system for impending pathologic fractures. Clin. Orthop. Relat. Res..

[11] Mac Niocaill R.F., Quinlan J.F., Stapleton R.D., Hurson B., Dudeney S., O’Toole G.C. (2011). Inter- and intra-observer variability associated with the use of the Mirels’ scoring system for metastatic bone lesions. Int. Orthop..

[12] Howard E.L., Shepherd K.L., Cribb G., Cool P. (2018). The validity of the Mirels score for predicting impending pathological fractures of the lower limb. Bone Jt. J..

[13] Evans A.R., Bottros J., Grant W., Chen B.Y., Damron T.A. (2008). Mirels’ rating for humerus lesions is both reproducible and valid. Clin. Orthop. Relat. Res..

[14] Hoban K.A., Downie S., Adamson D.J.A., MacLean J.G., Cool P., Jariwala A.C. (2022). Mirels’ score for upper limb metastatic lesions: Do we need a different cutoff for recommending prophylactic fixation?. JSES Int..

[15] Damron T.A., Nazarian A., Entezari V., Brown C., Grant W., Calderon N., Zurakowski D., Terek R.M., Anderson M.E., Cheng E.Y. (2016). CT-based Structural Rigidity Analysis Is More Accurate than Mirels Scoring for Fracture Prediction in Metastatic Femoral Lesions. Clin. Orthop. Relat. Res..

[16] Riaz S., Bashir H., Niazi I.K., Butt S., Qamar F. (2018). 99mTc MDP SPECT-CT-Based Modified Mirels Classification for Evaluation of Risk of Fracture in Skeletal Metastasis: A Pilot Study. Clin. Nucl. Med..

[17] Kaupp S.M., Mann K.A., Miller M.A., Damron T.A. (2021). Predicting Fracture Risk in Patients with Metastatic Bone Disease of the Femur: A Pictorial Review Using Three Different Techniques. Adv. Orthop..

[18] Toci G.R., Bressner J.A., Morris C.D., Fayad L., Levin A.S. (2021). Can a Novel Scoring System Improve on the Mirels Score in Predicting the Fracture Risk in Patients with Multiple Myeloma?. Clin. Orthop. Relat. Res..

[19] Crenn V., Carlier C., Gouin F., Sailhan F., Bonnevialle P. (2020). High rate of fracture in long-bone metastasis: Proposal for an improved Mirels predictive score. Orthop. Traumatol. Surg. Res..

[20] Amendola R.L., Miller M.A., Kaupp S.M., Cleary R.J., Damron T.A., Mann K.A. (2023). Modification to Mirels scoring system location component improves fracture prediction for metastatic disease of the proximal femur. BMC Musculoskelet. Disord..

[21] Desai V.S., Amendola R.L., Mann K.A., Damron T.A. (2024). Internal validation of modified Mirels’ scoring system for pathologic femur fractures. BMC Musculoskelet. Disord..

